# A New Driver for Lupus Pathogenesis is conserved in Humans and Mice

**DOI:** 10.35248/2684-1630.19.4.144

**Published:** 2019-11-21

**Authors:** Adam C Pagenkopf, Yun Liang

**Affiliations:** Department of Medical Microbiology and Immunology, University of Wisconsin, Wisconsin, USA

## INTRODUCTION

Lupus is experienced as an “aggressive and expansive” disease affecting patients’ activities of daily living, sense of self, and social functioning [1]. Currently there is no cure. Despite substantial efforts, the lupus drug development field has witnessed only one FDA-approved therapy in the last 50 years. There is an urgent need to better our understanding of the pathogenic mechanisms for lupus, and to develop novel therapeutic strategies for both cutaneous and systemic lupus erythematosus.

## FINDINGS

Like many autoimmune diseases, one of the most notable features of systemic lupus erythematosus is its greater prevalence in females than males. Therefore, it has been hypothesized that differences in gene expression and regulation between may underlie sex-biased autoimmunity including lupus [2,3].

Indeed, a female-biased gene network has been described in human skin which associates significantly with susceptibility to autoimmunity. This network is downstream of a putative transcription factor, VGLL3 (‘vestigial family member 3’), which is also female biased [4]. Genome-wide, VGLL3 targets are enriched for genes dysregulated in lupus, scleroderma, and Sjögren’s syndrome. These results identified the VGLL3 pathway as a previously unknown promoter of female-biased autoimmunity.

Building on this discovery, a recent study examines the immunological function of VGLL3 in mice and describes a novel mouse model that recapitulates many aspects of human cutaneous and systemic lupus [5]. Mouse and human VGLL3 exhibit 87% sequence homology. Wild-type mice exhibited a conserved sex-bias in VGLL3 expression in skin; the mice’s mean mRNA level of endogenous VGLL3 was found to be 3-fold higher in females.

The transgenic mouse model was generated by introducing a construct that produced murine VGLL3 under the control of the bovine keratin K5 promoter, which has previously been shown to drive expression primarily in the basal compartment and epithelia of the skin and secondarily in other stratified epithelia [6]. Overexpression of VGLL3 was confirmed by immunofluorescence staining, whereby increased VGLL3 staining was observed in the epithelial layer of skin.

The mouse model exhibited gross and microscopic similarities to cutaneous human lupus. Lesions, skin thickening, and scaling developed at 6–12 weeks of age. These features were preferentially located towards the ears and face, sites that are commonly associated with human cutaneous lupus. Microscopic examination of mouse lesional skin using simple hematoxylin-eosin and periodic acid-Schiff techniques revealed more similarities to human lupus lesions. These microscopic features included neutrophil infiltration and exocytosis in the dermis, thickening of the squamous epidermis, accumulation of melanin in the upper dermis, formation of vacuoles in basal cells, apoptotic keratinocytes, and basement membrane thickening.

Immunological analysis of transgenic inflamed skin tissue was in concordance with that observed in lupus patients and accepted mouse models of lupus. Immune complex deposition was observed by immunofluorescence in skin tissue. Immunohistochemistry revealed an inflammatory infiltrate that was enriched with markers for T cells; B cells dendritic cells, neutrophils, and macrophages. Flow cytometry analysis of skin associated cells showed B cell, plasmacytoid dendritic cell, and neutrophil expansion.

Gene expression in the transgenic and control skin was analyzed revealing similarities to expression patterns seen in lupus patients. Significantly increased mRNA expression of eleven inflammatory and lupus-related markers was observed in the skin of VGLL3 overexpressing mice compared to wild type mice. Several of these were confirmed by immunofluorescence. A broader RNA-Seq approach analyzing 811 genes in the same skin sites revealed a shared pattern in differential gene expression compared to that observed in orthologous genes dysregulated in the skin of discoid lupus and subacute cutaneous lupus patients.

Overexpression of VGLL3 in skin led to the development of systemic inflammation in the mouse model. Skin-draining lymph nodes and spleen mean mass was swollen by 4-fold and 2-fold, respectively. Mass cytometry analysis of the skin-draining lymph nodes and spleen showed significantly expanded B-cell populations. Significant increases in autoantibody production were detected in the serum by indirect immunofluorescence and enzyme-linked immunosorbent assay. Peripheral blood mononuclear cells in the mice displayed altered gene expression which, across 477 genes, was significantly associated with the pattern of orthologous genes dysregulated in patients with systemic lupus.

The transgenic mice’s skin symptoms were frequently severe enough to warrant euthanasia at 4–5 months of age, rendering certain types of studies, particularly regarding kidney damage, impracticable. Despite this, subtle inflammation of the glomeruli could be observed through histology. Immune complexes, an early marker of lupus nephritis, were also detected in the glomeruli by immunofluorescence.

The lupus-like phenotypes in the Vgll3-transgenic mice raise the question whether VGLL3 also sensitizes skin to UV radiation. In lupus-related photoprovocation an above threshold dose of UV irradiation, which only penetrates the deeper dermis, can lead to systemic autoimmune symptoms over a period of days or even weeks after an event [7,8]. It is experienced by 40%–90% of lupus patients [8] and recognized as one the more life-altering symptoms of the disease, most strikingly affecting patients’ daily functioning [9]. Future study on UV sensitivity of VGLL3-overexpressing mice may lead to a better understanding of the mechanisms underlying photosensitivity in lupus and how environmental factors, including UV radiation, at the site of epithelial surfaces provide the inciting “first hit” in the break in self-tolerance.

## CONCLUSION

In conclusion, the findings from the new mouse model together with the previous report supports the continued focus on the skin-immune system interface in lupus. Taken together with previous findings, VGLL3 represents a female-biased factor that can drive development of cutaneous and systemic lupus.
